# Investigating multisite pain as a predictor of self-reported falls and falls requiring health care use in an older population: A prospective cohort study

**DOI:** 10.1371/journal.pone.0226268

**Published:** 2019-12-11

**Authors:** Victoria K. Welsh, Christian D. Mallen, Reuben Ogollah, Ross Wilkie, John McBeth

**Affiliations:** 1 Arthritis Research UK Primary Care Centre, Research Institute for Primary Care & Health Sciences, Keele University, Staffordshire, United Kingdom; 2 Faculty of Medicine & Health Sciences, South Block, Queen’s Medical Centre, Nottingham, United Kingdom; 3 Arthritis Research UK Centre for Epidemiology, Faculty of Biology, Medicine and Health, The University of Manchester, Manchester, United Kingdom; University of Malaya, MALAYSIA

## Abstract

Older people are continuing to fall despite fall prevention guidelines targeting known falls’ risk factors. Multisite pain is a potential novel falls’ risk factor requiring further exploration. This study hypothesises that: (1) an increasing number of pain sites and widespread pain predicts self-reported falls and falls recorded in primary and secondary healthcare records; (2) those relationships are independent of known falls’ risk factors and putative confounders. This prospective cohort study linked data from self-completed questionnaires, primary care electronic health records, secondary care admission statistics and national mortality data. Between 2002–2005, self-completion questionnaires were mailed to community-dwelling individuals aged 50 years and older registered with one of eight general practices in North Staffordshire, UK(n = 26,129) yielding 18,497 respondents. 11,375 respondents entered the study; 4386 completed six year follow-up. Self-reported falls were extracted from three and six year follow-up questionnaires. Falls requiring healthcare were extracted from routinely collected primary and secondary healthcare data. Increasing number of pain sites increased odds of future 3 year (odds ratio 1.12 (95% confidence interval: 1.01–1.24)) and 6 year self-reported fall (odds ratio 1.02 (1.00–1.03)) and increased hazard of future fall requiring primary healthcare (hazard ratio 1.01 (1.00–1.03)). The presence of widespread pain increased odds of future 3 year (odds ratio 1.27 (0.92–1.75)) and 6 year fall (odds ratio 1.43(1.06–1.95)) and increased hazard of future fall requiring primary healthcare (hazard ratio 1.27(0.98–1.65)). Multisite pain was not associated with future fall requiring secondary care admission. Multisite pain must be included as a falls’ risk factor in guidelines to ensure clinicians identify their older patients at risk of falls and employ timely implementation of current falls prevention strategies.

## Introduction

Despite a myriad of international falls prevention guidelines targeting known fall’s risk factors [1,2,3], falls remain a common experience of ageing with worldwide; 12 month prevalence estimates for falls in older people approximately 20% [4,5,6]. Falls prevalence and their associated burden is set to increase with global population ageing; the number of older adult fatal falls in the US is projected to reach 100,000 annually by 2030 with a corresponding increased estimated annual cost of $100 billion to the US healthcare system [7]. Multisite pain (pain in more than one part of the body) has been proposed as a novel falls’ risk factor; older adults reporting multisite pain were found to have an increased risk of future self-reported fall at 18 months [8]. Subsequent studies have found similar results when measuring the risk of multisite pain and self-reported fall in the previous 12 months [9,10,11]. The bias associated with self-reporting of falls [12] and the dearth of studies exploring the relationship between multisite pain and falls requiring primary healthcare use or hospitalisation mean further research is required to explore the relationship between multisite pain and falls of all severity to enable this potential falls’ risk factor to be included in updated falls prevention strategies internationally.

Using a combination of self-report and routinely collected healthcare data, this study tests the hypotheses that within a community-dwelling population of older people: (1) an increasing number of pain sites and widespread pain would predict self-reported falls and falls recorded in primary and secondary healthcare records; and (2) those relationships would be independent of known falls risk factors that may act as confounders.

## Materials and methods

### Study design and participants

**Fig 1:**
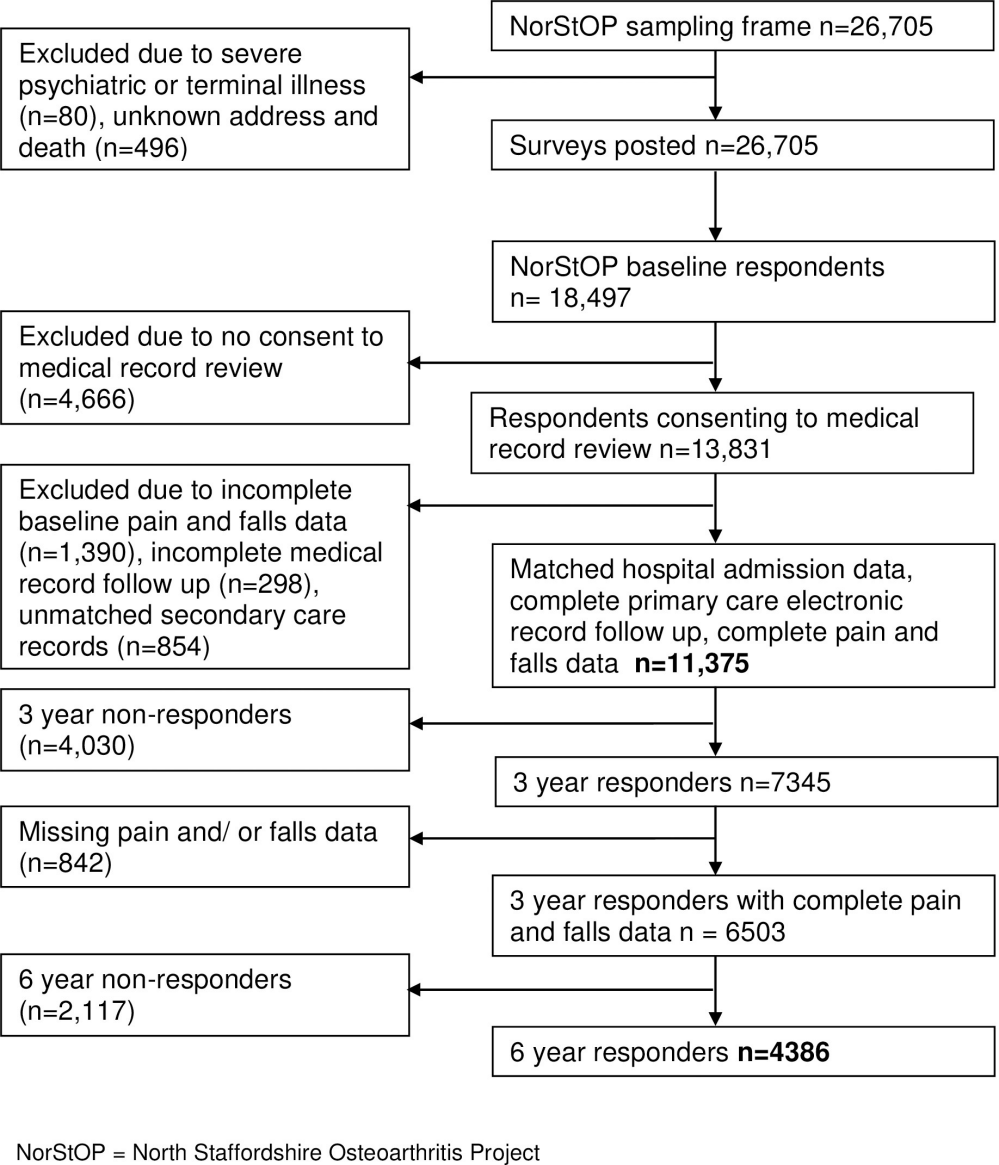
Study sample derivation from sampling frame through 6 year follow up.

The study linked self-completed questionnaire data from an existing prospective cohort study called the North Staffordshire Osteoarthritis Project (NorStOP) [13], with routinely collected primary and secondary health care records and mortality data. Fig 1 outlines study sample derivation. Permission to undertake data linkage was obtained from the Secretary of State for Health under Section 251 of the National Health Service Act 2006 (National Information Governance Board for Health and Social Care: Ethics and Confidentiality Committee 8-02(FT1)/2012). The study was approved by the National Research Ethics Service Committee West Midlands: Staffordshire (12/WM/0200).

#### The NorStOP cohort

The NorStOP sampling frame comprised all individuals aged 50 years and older who were registered with one of eight general practices in North Staffordshire, United Kingdom (n = 26,705). Self-completion questionnaires were mailed to the eligible population (n = 26,129) in three recruitment waves between April 2002 and April 2005. Responders to the baseline postal survey (n = 18,497) were mailed out further surveys at three and six years.

#### Routinely collected data from primary and secondary electronic health records

In the United Kingdom, consultations within a primary care setting are routinely recorded within primary care electronic health records using standardised Read Codes [14] (a coded thesaurus of clinical terms [15]) and accompanying free-text; prescribing information is also recorded. Hospital Episode Statistics (HES) data, a comprehensive record-based system that contains identifiable patient information from all National Health Service (NHS) Trusts in England [16] uses the International Classification of Disease version 10 to record diagnosis-related information. The HES Admitted Patient Care dataset was used to capture information about falls that required inpatient admission. The Office for National Statistics dataset contains identifiable patient information on cause of death as detailed on the death certificate [17].

#### Data linkage

NorStOP responders at baseline who consented to medical record review (n = 13,831) had their primary care electronic health record, HES admission data and mortality data linked to baseline, 3 year and 6 year survey responses by sending individual level identifiable information (NHS number, postcode, date of birth, sex) and a unique pseudo-identifier to the NHS Information Centre (now NHS Digital). Linked records were returned with the pseudo-identifier to the data custodian; 854 respondents had their records returned unmatched.

### Variables, data sources and measurement

#### Multisite pain

Participants were asked in the baseline questionnaire if they had ‘experienced any pain lasting at least one day, during the past month’; ‘yes’ or ‘no’ responses were recorded. Pain included ‘‘any ache, discomfort or stiffness” and excluded pain caused by feverish illness or menses. Respondents answering ‘yes’ were asked to indicate their pain site(s) on a body manikin that identified 44 discrete non-overlapping anatomical areas [18]. The number of pain sites equalled the number of discrete shaded areas on the manikin. Pain pattern was categorised according to widespreadness. ‘Widespread pain was defined as pain identified in the axial skeleton plus pain above and below the waist plus left and right-sided pain’ [19,20]. Respondents indicating pain that did not fulfil widespread pain criteria were categorised as ‘some pain’; respondents indicating no pain were categorised accordingly.

#### Falls

Self-reported falls data was extracted from survey responses. Respondents were asked at baseline, three and six year follow-up if they had ‘suffered from a fall or falls in the past three months’; yes or no responses were recorded. Data on falls requiring primary healthcare was extracted from primary care electronic health records using Read Codes relating to falls extracted from the NHS Clinical Terminology Browser and confirmed by a group of 17 practising general practitioners; Read Codes included ‘16D..’, ‘U10..’, ‘TC…’ (S1 Table contains the complete list of Read Codes used). Data on falls requiring secondary care admission were extracted from HES Admitted Patient Care using the International Classification of Disease version 10 codes W01, W05, W06, W07, W08, W10, W17, W18 and W19.

#### Potential confounders and effect modifiers: Covariates

Covariates were selected following a literature review of falls risk factors and consideration of covariates that had been included in publications identified in a systematic review and meta-analysis investigating multisite pain and falls [21]. The details of the health survey used in the NorStOP cohort studies have been published previously [13].

Socio-demographic characteristics: Participants’ age and sex were obtained from primary care electronic health records. Age was treated as a continuous variable and dichotomised into adults aged 50–64 years and adults aged 65 years and older. Socio-economic status was determined using self-reported educational attainment (further education beyond aged 16 years), self-reported occupational status (according to the Office for National Statistics categories [22,23]) and self-reported income adequacy or inadequacy to measure individual status over the life course [24]. The Index of Multiple Deprivation was used to measure area-level socio-economic status [25].

Physical health measures: Multimorbidity was measured using the Charlson Comorbidity Index, a measure that takes into account the number and seriousness of co-morbid conditions to predict all-cause mortality at one year [26,27]. The Charlson Comorbidity Index has been validated in different populations and has been shown to be a valid summary co-morbidity measure for use in epidemiological studies to predict outcome [28]. Each respondent is scored according to the presence of the listed diagnostic categories as indicated by Read Codes in the primary care electronic health records. The Charlson Comorbidity Index scores range from 0–33; the highest score in the study sample was 8. The measure was treated categorically, with categories of 0, 1–2 and 3–8 respectively dichotomized at the mean value excluding zero. Vision, hearing and dizziness were measured using the baseline NorStOP survey responses to dichotomously self-reported problems over the past three months with ‘eyesight (excluding the need for glasses)’, ‘deafness’, and ‘dizziness or unsteadiness’. Body mass index was calculated using self-reported height and weight from the baseline NorStOP survey. Body mass index was included in analyses as a continuous variable, and categorised according to the World Health Organisation [29] as below 18.5 (underweight), 18.5–24.9 (normal weight), 25.0–29.9 (pre-obesity), 30.0–34.9 (obesity class I), 35.0–39.9 (obesity class II) and 40 or greater (obesity class III) to describe study participant characteristics.

Mental health measures: Anxiety and depression were measured using the Hospital Anxiety and Depression Scale [30]. The Hospital Anxiety and Depression Scale is a valid measure for anxiety and depression case finding, and for assessment of symptom severity; it also performs well in a primary care setting [31]. The Hospital Anxiety and Depression Scale was used as a continuous measure in analysis (range 0–21). A categorical measure to describe study participant characteristics used cut off points according to the literature [32] of 0–7 (no clinical anxiety or depression), 8–10 (borderline) and 11 or over (clinical caseness of anxiety or depression). The NorStOP survey used the alertness behavioural subscale of the Sickness Impact Profile [33] to measure subjective cognitive complaint. The scale asks questions about multitasking, minor accidents, reaction times, task completion, problem solving, orientation, forgetfulness, attention span, mistakes and concentration skills. Each question is weighted and summed to provide an overall score from 0 to 100. The score was analysed on a continuous scale and as categorical data to describe participant characteristics. The categories were 0 (no cognitive complaint), 1–14 (mild cognitive complaint), 15–38 (moderate cognitive complaint), and 39–100 (severe cognitive complaint); categories were generated based on 33% of participants in each non-zero category, as used in published research [34]. Although not measuring cognitive impairment, recorded difficulties with each component of the alertness behavioural subscale of the Sickness Impact Profile score is indicative of cognitive impairment, defined as the ‘symptomatic pre-dementia stage on the continuum of cognitive decline, characterised by objective impairment in cognition that is not severe enough to require help with usual activities of daily living’ [35].

Medication covariates: Prescription information for the three months prior to baseline survey distribution was extracted from primary care electronic health records. Total medication count, measured continuously, was derived from the number of different British National Formulary sub-chapter codes [36]. Analgesic use was categorised according to published research as (0) no analgesics, (1) basic analgesics (paracetamol), (2) weak combination opioids, (3) moderate combination opioids and opioids, (4) strong combination opioids and opioids, (5) very strong single opioids [37]; the highest numerical category was taken to represent analgesic use in respondents. Non-steroidal anti-inflammatory drug use (NSAID) was recorded dichotomously.

Physical functioning: Physical functioning is measured in the NorStOP survey using the Medical Outcomes Study SF-36 Physical Functioning subscale, with ten separate items and an overall component score [13, 38]. The single item “Does your health limit you in walking 100 yards?” was used to measure physical functioning with answers categorised as ‘no, not limited’, ‘yes, limited a little’, and ‘yes, limited a lot’. The single item was used as this has been found to measure the most severe level of mobility limitation, previous research has used this single item to measure mobility limitation [39], and it has been suggested that combining items from the physical functioning subscale of the Medical Outcomes Study SF-36 may be mathematically flawed [40,41,42].

### Statistical analysis

Self-reported falls period prevalence was estimated over the three months prior to baseline survey return. Period prevalence of falls requiring primary healthcare use or secondary healthcare (hospital) admission was measured from the start of respondents’ corresponding baseline survey mail out until the end of the six year follow-up for respondents who completed all follow-up surveys. For respondents who did not complete three year follow-up, their study period ended at the end of the corresponding three year survey mail out period. The number of respondents who had ever had a fall recorded in their primary care electronic health record or secondary care admission data was used as the numerator.

Logistic regression was used to examine the association between multisite pain and self-reported falls amongst respondents with complete follow-up (n = 4386). Pain was modelled as either number of pain sites or widespreadness. Multivariable logistic regression models examined the odds of self-reporting a future fall at three or six years associated with baseline pain status whilst taking account of baseline covariate measures. Step-wise models were built, starting with the unadjusted model and then adding in covariate groups (socio-demographics, physical health measures, mental health measures, medication, physical functioning, and history of baseline self-reported fall) until all covariates were included in the model.

All variables were maintained in the model to maximise clinical relevance; excluding non-statistically significant variables would reduce clinical applicability. All covariates were interacted with pain. All socio-demographic covariates were interacted with physical health, mental health, medication and physical functioning. All physical health covariates were interacted with all mental health, medication and the physical functioning covariate. All mental health covariates were interacted with all medication and the physical functioning covariate. All medication covariates were interacted with the physical functioning covariate. Interaction terms with p<0.05 on testing were added to the full model and the likelihood ratio test used to compare the model containing the interaction terms and the model with no interaction terms. A likelihood ratio test yielding a p-value <0.05 indicated the model containing the interaction terms must be used to draw conclusions.

Cox proportional hazards models tested the association between multisite pain and future falls requiring primary healthcare use and falls requiring hospital admission. Pain was entered into the model as either the number of pain sites or widespreadness. Univariable associations were first tested using log-rank test for equality of survivor functions and Kaplan-Meier curve generation to assess time to first fall event for categorical data; Cox proportional hazard models with a single predictor variable was used for continuous data. All covariates were added to the Cox proportional hazard model using the stepwise approach described for the logistic regression; interactions and likelihood ratio testing were applied as described. Proportionality assumption for Cox proportional hazard models was checked using Schoenfeld residuals and by including a time-dependent variable by time interaction in the model. Stata 14 (Statacorp, College Station, Texas) was used for analysis.

A sensitivity analysis was performed to investigate the relationship between multisite pain, and future falls requiring primary healthcare and hospital admission within the sample with complete follow up (n = 4,386), the sample less susceptible to missing data.

## Results

The baseline sample contained 11,375 respondents; 4386 had complete baseline pain and falls data and completed six year follow-up (Fig 1). Table 1 presents baseline sample study characteristics according to baseline pain status. The sample completing follow-up (n = 4,386) were younger, lived in less deprived areas, had less depression (there was no difference in anxiety levels) and physical morbidity, fewer medications and strong analgesics prescribed, and were less limited in physical functioning than the baseline sample (p<0.05). At baseline, 26.6% (n = 3026) of respondents reported no pain, 5.3% (n = 605) reported single site pain, 68.1% (n = 7744) reported pain in two or more sites and 26.9% (3062) met the criteria for widespread pain; the median number of pain sites was 4 (interquartile range 0–9). 12.5% (1417) respondents self-reported a fall in the baseline survey, 14.4% (1056) in the three year follow-up survey and 14.2% (685) in the six year follow-up survey. 783 (6.9%) respondents had at least one fall requiring primary healthcare use and 804 (7.1%) respondents had at least one fall requiring hospital admission.

**Table 1:** Respondent characteristics in baseline study sample according to baseline pain status.

Variable	Number of pain sites Mean (95% CI)		No pain n = 3138 (%)	Some pain n = 5175 (%)	Widespread pain n = 3062 (%)
Age, years Mean (95% CI)	50–54	5.9 (5.5–6.2)	444 (27.7)	720 (44.9)	441 (27.5)
	55–59	6.3 (6.0–6.6)	588 (27.8)	905 (42.9)	619 (29.3)
	60–64	6.4 (6.0–6.7)	457 (25.8)	821 (46.4)	493 (27.8)
	65–69	6.1 (5.8–6.4)	475 (26.1)	838 (46.1)	505 (27.8)
	70–74	5.6 (5.3–6.0)	460 (30.2)	690 (45.4)	371 (24.4)
	75–79	6.2 (5.8–6.6)	357 (27.8)	596 (46.4)	332 (25.8)
	80–84	6.2 (5.7–6.7)	223 (27.2)	384 (46.8)	214 (26.1)
	85–89	5.7 (5.0–6.5)	99 (30.5)	163 (50.2)	63 (19.4)
	90–99	5.8 (4.5–7.1)	35 (30.0)	58 (49.6)	24 (20.5)
Sex					
Male (n, %)	5.4 (5.3–5.6)		1575 (29.9)	2459 (46.7)	1229 (23.3)
Female (n, %)	6.7 (6.5–6.9)		1563 (25.6)	2716 (44.4)	1833 (30.0)
Education > 16y n = 11,175					
Yes	5.2 (4.8–5.5)		465 (33.7)	613 (44.4)	304 (22.0)
No	6.2 (6.1–6.4)		2623 (26.8)	4476 (45.7)	2694 (27.5)
Missing	6.6 (5.6–7.4)		50 (25.0)	86 (43.0)	64 (32.0)
Occupational class n = 10,543					
Manual	6.3 (6.1–6.5)		1669 (26.1)	2881 (45.1)	1839 (28.8)
Non-manual	5.6 (5.4–5.8)		1274 (30.7)	1903 (45.8)	977 (23.5)
Unknown	7.0 (6.5–7.6)		195 (23.4)	391 (47.0)	246 (29.6)
Income adequacy n = 11,178					
Adequate	4.9 (4.8–5.1)		2022 (32.0)	2941 (46.5)	1357 (21.5)
Inadequate	7.5 (7.3–3.8)		1074 (22.1)	2143 (44.1)	1641 (33.8)
Unknown	7.2 (6.1–8.3)		42 (21.3)	91 (46.2)	64 (32.5)
IMD n = 11,372					
Least deprived	5.2 (4.9–5.4)		700 (30.5)	1089 (47.5)	506 (22.1)
2^nd^ least dep.	5.5 (5.2–5.8)		704 (29.9)	1078 (45.9)	569 (24.2)
Mid deprived	6.0 (5.7–6.3)		618 (27.3)	1044 (46.1)	603 (26.6)
2^nd^ most dep.	6.6 (6.3–6.9)		587 (26.6)	982 (44.4)	642 (29.0)
Most deprived	7.2 (6.9–7.6)		528 (23.5)	981 (43.6)	741 (32.9)
Missing	2.3 (-5.9–13.9)		1 (33.3)	1 (33.3)	1 (33.3)
Multimorbidity CCI					
0	5.6 (5.5–5.8)		2461 (29.1)	3851 (45.5)	2159 (25.5)
1	7.2 (6.9–7.6)		443 (23.3)	875 (46.1)	581 (30.6)
2	7.8 (7.3–8.4)		234 (23.3)	449 (44.7)	322 (32.0)
BMI n = 10959					
Underweight	6.5 (5.2–7.7)		43 (27.4)	70 (44.6)	44 (28.0)
Normal weight	5.1 (4.9–5.3)		1373 (33.0)	1872 (45.0)	911 (21.9)
Pre-obesity	6.1 (5.9–6.3)		1231 (27.1)	2086 (46.0)	1227 (27.0)
Obesity class I	7.8 (7.5–8.2)		313 (20.4)	713 (46.4)	510 (33.2)
Obesity class II	9.2 (8.4–10.0)		53 (12.9)	178 (43.3)	180 (43.8)
Obesity class III	11.3 (9.8–12.8)		17 (11.0)	63 (40.7)	75 (48.4)
Missing	6.4 (5.6–7.1)		108 (26.0)	193 (46.4)	115 (27.6)
Continuous					
Mean			25.7	26.7	27.6
range			14.4–60.2	13.8–70.3	11.9–71.5
Hearing problem					
Yes	7.3 (6.9–7.6)		435 (20.7)	994 (47.3)	673 (32.0)
No	5.8 (6.7–6.0)		2703 (29.2)	4181 (45.1)	2389 (25.8)
Vision problem					
Yes	7.8 (7.4–8.1)		518 (22.0)	1036 (44.0)	805 (34.1)
No	5.7 (5.5–5.8)		2620 (29.0)	4139 (45.9)	2257 (25.0)
Anxiety score n = 11110					
0–7	4.6 (4.5–4.8)		2367 (34.6)	3149 (46.0)	1335 (19.5)
8–10	7.4 (7.1–7.7)		447 (19.2)	1104 (47.3)	781 (33.5)
11+	9.8 (9.4–10.2)		324 (14.8)	922 (42.1)	946 (43.2)
Missing	6.4 (5.5–7.4)		61 (23.0)	132 (49.8)	72 (27.2)
Continuous					
Mean score			5.2 (0–21)	6.6 (0–21)	8.4 (0–21)
SD			SD 3.8	SD 4.0	SD 4.3
Depression					
n = 11120					
0–7	5.0 (4.9–5.2)		2724 (31.3)	4053 (46.5)	1934 (22.2)
8–10	9.1 (8.7–9.5)		241 (15.9)	646 (42.7)	625 (41.3)
11+	11.4 (10.8–12.0)		113 (12.6)	350 (39.0)	434 (48.4)
Missing	6.2 (5.3–7.2)		60 (23.5)	126 (49.4)	69 (27.1)
Continuous					
Mean score			3.4 (0–21)	4.7 (0–21)	6.3 (0–21)
SD			SD 3.2	SD = 3.5	SD = 3.9
Cognitive complaint n = 10774					
No	4.3 (4.2–4.5)		2114 (35.7)	2712 (45.8)	1100 (18.6)
Mild	6.9 (6.6–7.3)		353 (21.8)	764 (47.2)	503 (31.1)
Moderate	7.9 (7.5–8.2)		315 (18.4)	772 (45.1)	624 (36.5)
Severe	9.9 (9.4–10.4)		236 (15.6)	649 (42.8)	632 (41.7)
Missing	7.1 (6.4–7.7)		120 (20.0)	278 (46.3)	203 (33.8)
Continuous					
Mean (range)			8.6 (0–100)	14.0 (0–100)	22.3 (0–100)
SD			18.2	22.5	27.4
Total medication					
0 meds	3.8 (3.6–3.9)		1015 (39.2)	1140 (44.1)	432 (16.7)
1–2 meds	5.0 (4.8–5.2)		861 (29.8)	1394 (48.3)	633 (21.9)
3–4 meds	6.0 (6.8–6.4)		633 (27.6)	1012 (44.0)	653 (28.4)
5–7 meds	7.8 (7.5–8.1)		403 (19.3)	989 (47.3)	700 (33.5)
8+meds	9.9 (9.4–10.3)		226 (15.0)	640 (42.4)	644 (42.7)
Continuous					
Mean			2.5(2.4–2.6)	3.4(3.4–3.6)	4.6 (4.5–4.8)
SD			2.8	3.4	3.9
Pain medication					
0	4.6 (4.5–4.8)		2838 (34.5)	3696 (44.9)	1697 (20.6)
1	7.0 (6.5–7.5)		163 (18.8)	433 (49.9)	271 (31.3)
2	10.2 (9.6–10.9)		55 (7.1)	376 (48.7)	341 (44.2)
3	10.6 (10.0–11.2)		53 (6.8)	433 (45.9)	369 (47.3)
4 and 5	12.2 (11.5–12.9)		29 (4.0)	312 (43.0)	384 (53.0)
NSAID use					
Yes	10.3 (9.8–10.8)		75 (6.1)	596 (48.3)	562 (45.6)
No	5.6 (5.5–5.7)		3063 (30.2)	4579 (45.2)	2500 (24.7)
Physical functioning (difficulty walking 100 yards) n = 11172					
Yes, a lot	11.7 (11.3–12.3)		130 (9.4)	593 (43.0)	655 (47.5)
Yes, a little	9.2 (8.6–9.6)		236 (12.6)	850 (45.3)	790 (42.1)
No	4.4 (4.3–4.5)		2714 (34.3)	3682 (45.9)	1572 (19.9)
Missing	5.2 (4.2–6.1)		58 (28.6)	100 (49.3)	45 (22.2)

95% CI = 95% confidence interval; NPS = number of pain sites; IMD = Index of multiple deprivation; Education > 16y = continuing in full time education beyond aged 16 years; dep. = deprived; CCI = Charlson Comorbidity Index: 0 = no CCI morbidities, 1 = 1 CCI morbidity, 2 = 2–8 CCI morbidities; BMI = body mass index; anxiety and depression scale scores: 0–7 = normal, 8–10 = borderline, 11 or over = clinical 'caseness'; Pain medication maximum category: 0 No analgesics, 1 Basic analgesics, 2 Weak combination opioids, 3 Moderate combination opioids and opioids, 4 Strong combination opioids and opioids, 5 Very strong single opioids; SD = standard deviation; NSAID = non-steroidal anti-inflammatory drugs. Age groups 90–99 combined due to small numbers in cells (<5); n = 11,375 unless otherwise stated.

Table 2 presents the unadjusted odds ratios (OR) and hazard ratios (HR) (with 95% confidence intervals) for each fall classification. Table 3 presents the adjusted OR and HR (with 95% CIs) for each fall classification according to the number of pain sites and Table 4 presents adjusted OR and HR (with 95% CIs) for each fall classification according to widespread pain status.

**Table 2:** Unadjusted odds ratios and hazard ratios for multisite pain and self-reported falls, falls requiring primary healthcare utilisation or secondary healthcare admission.

Pain measure	3 year self reported fall n = 4,386	6 year self reported fall n = 4,386	Fall requiring primary healthcare n = 11,375	Fall requiring hospital admission n = 11,375
Number of pain sites	1.06 (1.05–1.07) p<0.001	1.06 (1.05–1.07) p<0.001	1.02 (1.02–1.03) p<0.001	1.01 (1.00–1.02) p = 0.062
Widespread measure: Some pain	1.55 (1.21–1.99) p = 0.001	1.62 (1.28–2.06) p<0.001	1.37 (1.14–1.65) p = 0.001	0.96 (0.81–1.13) p = 0.611
Widespread measure: Widespread pain	2.96 (2.31–3.80) p<0.001	2.90 (2.27–3.70) p<0.001	1.51 (1.24–1.84) p<0.001	1.09 (0.91–1.31) p = 0.357

**Table 3:** Adjusted odds ratios and hazard ratios for number of pain sites and self-reported falls, falls requiring primary health care utilisation or secondary health care admission.

Covariate	3 year self reported fall n = 3,801 OR (95% CI)	6 year self reported fall n = 3,801 OR (95% CI)	Falls requiring primary health care n = 9,234 HR (95% CI)	Falls requiring hospital admission n = 9,234 HR (95% CI)
Number of pain sites	1.12 (1.01–1.24) p = 0.037	1.02 (1.00–1.03) p = 0.035	(1.00–1.02) p = 0.106	1.00 (0.99–1.01) p = 0.938
Age (years)	1.04(1.02–1.05) p<0.001	1.03 (1.02–1.05) p<0.001	1.07 (1.06–1.08) p<0.001	1.07 (1.06–1.08) p<0.001
Sex: Male	0.83 (0.66–1.04) p = 0.097	0.84 (0.68–1.04) p = 0.102	0.53 (0.44–0.63) p<0.001	0.57 (0.47–0.68) p<0.001
FT Ed >16y: No	1.04 (0.75–1.43) p = 0.829	0.76 (0.59–1.02) p = 0.073	0.90 (0.69–1.16) p = 0.434	0.99 (0.75–1.31) p = 0.962
Income adequate	1.08 (0.86–1.35) p = 0.526	0.79 (0.64–0.98) p = 0.028	1.01 (0.85–1.20) p = 0.936	1.13 (0.95–1.34) p = 0.173
Occ Class non-manual	0.62(0.46–0.85) p = 0.003	0.94 (0.76–1.16) p = 0.541	1.09 (0.91–1.30) p = 0.342	0.99 (0.83–1.18) p = 0.898
IMD				
1)least dep.	Referent	Referent	Referent	Referent
2) 2^nd^ least	0.94 (0.67–1.30) p = 0.692	1.32 (0.98–1.78) p = 0.070	(0.78–1.30) p = 0.940	1.04 (0.79–1.39) p = 0.750
3)mid dep.	1.06 (0.76–1.47) p = 0.736	1.16 (0.85–1.58) p = 0.365	0.81 (0.63–1.06) p = 0.127	1.18 (0.88–1.59) p = 0.268
4) 2^nd^ most	1.18 (0.84–1.65) p = 0.342	1.05 (0.75–1.45) p = 0.789	0.87 (0.67–1.13) p = 0.304	1.19 (0.84–1.67) p = 0.329
5)most dep.	0.97 (0.68–1.38) p = 0.871	1.11 (0.80–1.55) p = 0.527	0.90 (0.69–1.17) p = 0.446	1.30 (0.88–1.94) p = 0.191
Dizzy: Yes	1.23 (0.96–1.58) p = 0.109	1.69 (1.34–2.13) p<0.001	1.20 (0.99–1.46) p = 0.067	1.09 (0.79–1.51) p = 0.587
Hearing deficit: Yes	0.91 (0.67–1.23) p = 0.529	0.85 (0.64–1.13) p = 0.269	0.86 (0.77–0.96) p = 0.006	1.00 (0.82–1.22) p = 0.986
Visual deficit: Yes	1.14 (0.86–1.49) p = 0.360	1.23 (0.95–1.59) p = 0.115	1.09 (0.99–1.21) p = 0.073	0.98 (0.81–1.19) p = 0.818
CCI score				
0	referent	Referent	Referent	Referent
1	0.75(0.55–1.03) p = 0.080	0.83 (0.62–1.12) p = 0.228	1.09 (0.88–1.36) p = 0.419	1.11 (0.83–1.50) p = 0.475
2–8	0.54 (0.33–0.88) p = 0.014	0.90 (0.59–1.36) p = 0.605	(0.79–1.38)p = 0.782	1.57 (0.99–2.49) p = 0.054
BMI	1.02 (1.00–1.04) p = 0.056	1.00 (0.98–1.02) p = 0.993	0.99 (0.98–1.00) p = 0.024	0.95 (0.92–0.98) p<0.001
Depression	1.01 (0.97–1.06) p = 0.554	1.03 (0.99–1.07) p = 0.205	1.02 (0.99–1.05) p = 0.256	1.03 (0.98–1.08) p = 0.225
Anxiety	1.02 (0.99–1.06) p = 0.231	0.98 (0.95–1.01) p = 0.254	0.98 (0.95–1.01) p = 0.177	0.99 (0.95–1.04) p = 0.742
Cognitive complaint	1.02 (1.01–1.02) p<0.001	1.01 (1.00–1.01) p = 0.001	1.00 (1.00–1.01) p = 0.064	1.00 (1.00–1.02) p = 0.115
Total medication	1.05 (1.00–1.09) p = 0.043	1.02 (0.98–1.06) p = 0.387	1.05 (1.02–1.08) p = 0.001	1.06 (1.00–1.11) p = 0.052
Analgesics				
None	Referent	Referent	Referent	Referent
Basic	1.16(0.76–1.79) p = 0.492	0.91 (0.60–1.37) p = 0.653	0.91 (0.68–1.22) p = 0.546	1.04 (0.80–1.37) p = 0.763
Weak op.	1.43 (0.89–2.30) p = 0.136	0.95 (0.62–1.47) p = 0.829	0.90 (0.65–1.24) p = 0.518	0.93 (0.68–1.27) p = 0.639
Mod. op.	1.54 (0.94–2.54)	0.95 (0.63–1.42)	1.20 (0.90–1.60)	0.84 (0.61–1.16)
	p = 0.086	p = 0.802	p = 0.222	p = 0.289
Strong op	2.34 (1.31–4.19) p = 0.004	1.23 (0.81–1.85) p = 0.327	(0.87–1.67)* p = 0.261*	1.41 (1.03–1.93)* p = 0.03*
V.strong op	2.39 (0.29–19.64) p = 0.415	0.44 (0.05–4.18) p = 0.478		
NSAIDs: Yes	0.81 (0.58–1.13) p = 0.217	1.58 (0.94–1.70) p = 0.114	1.08 (0.95–1.23) p = 0.247	0.91 (0.69–1.19) p = 0.486
Physical functioning				
No problem	Referent	Referent	Referent	Referent
A little	1.14 (0.84–1.55) p = 0.412	1.79 (1.35–2.38) p<0.001	(0.83–1.32) p = 0.691	0.46 (0.26–0.81) p = 0.007
A lot	1.13 (0.74–1.73) p = 0.560	1.84 (1.25–2.71) p = 0.002	0.80 (0.60–1.07) p = 0.128	0.17 (0.06–0.53) p = 0.002
Previous fall: Yes	2.60 (1.94–3.48) p<0.001	1.94 (1.45–2.59) p<0.001	1.51 (1.21–1.88) p<0.001	1.25 (0.89–1.74) p = 0.193

OR = odds ratios; HR = hazard ratios 95% CI = 95% confidence interval; FT Ed >16y = continuing in full time education beyond aged 16 years; IMD = Index of Multiple Deprivation divided into quintiles, where: least dep. = least deprived, 2nd least = 2nd least deprived, mid dep. = middle deprivation category, 2nd most = 2nd most deprived, most dep. = most deprived category. Charlson Comorbidity Index (CCI) score: 0 = no CCI comorbidities, 1 = 1 CCI comorbidity, 2–8 = 2 or more CCI comorbidities. BMI = body mass index. Total medication = total medication count. Analgesics: weak op. = weak opiates, mod. op. = moderate strength opiates, strong op. = strong opiates, v. strong op. = very strong opiates

* = for falls requiring primary health care and falls requiring hospitalisation, analgesic categories strong and very strong opioids are combined due to small numbers. NSAID = non steroidal anti = inflammatory drug. Physical functioning = ability to walk 100 yards: no problem = no physical limitation, a little = a little limitation, a lot = a lot of limitation in ability to walk 100 yards. Previous fall = baseline self-reported fall recorded as ‘yes’. Interaction terms with p<0.05 included in final models were: 3 year self-reported fall—‘NPSXmaximum analgesic category’, ‘NPSXoccupational class’, ‘NPSXage’; 6 year self-reported fall–none; falls requiring primary healthcare—‘NPSXage’, ‘NPSXcognitive complaint’, ‘NPSXdepression’, ‘NPSXmaximum analgesic category’, ‘NPSXNSAID use’, ‘NPSXphysical functioning’; falls requiring hospitalisation–none.

**Table 4:** Adjusted odds ratios and hazard ratios for widespread pain and self-reported falls, falls requiring primary health care utilisation or secondary health care admission.

Covariate	3 year self reported fall n = 3,801 OR (95% CI)	6 year self reported fall n = 3,801 OR (95% CI)	Falls requiring primary health care n = 9,234 HR (95% CI)	Falls requiring hospital admission n = 9,234 HR (95% CI)
No pain	Referent	Referent	Referent	Referent
Some pain	1.00 (0.75–1.33) p = 0.982	1.22 (0.93–1.61) p = 0.148	1.26 (1.01–1.57) p = 0.048	0.84 (0.69–1.04) p = 0.112
Widespread	1.27 (0.92–1.75) p = 0.143	1.43 (1.06–1.95) p = 0.021	1.27 (0.98–1.65) p = 0.094	0.98 (0.77–1.25) p = 0.907
Age (years)	(1.01–1.04) p = 0.002	1.03 (1.02–1.04) p<0.001	1.07 (1.06–1.08) p<0.001	1.07 (1.06–1.08) p<0.001
Sex: Male	0.83 (0.66–1.03) p = 0.094	0.84 (0.68–1.03) p = 0.092	0.52 (0.43–0.63) p<0.001	0.56 (0.47–0.68) p<0.001
FT Ed >16y: No	1.07 (0.78–1.47) p = 0.679	0.77 (0.59–1.02) p = 0.071	0.90 (0.69–1.17) p = 0.422	1.00 (0.76–1.32) p = 0.988
Income adequate	1.06 (0.84–1.33) p = 0.626	0.78 (0.63–0.97) p = 0.025	1.01 (0.84–1.20) p = 0.938	1.13 (0.95–1.34) p = 0.175
Occ Class non-manual	0.76 (0.61–0.95) p = 0.018	0.95 (0.77–1.17) p = 0.624	1.09 (0.92–1.31) p = 0.317	0.99 (0.83–1.18) p = 0.920
IMD				
1)least dep.	Referent	Referent	Referent	Referent
2) 2^nd^ least	0.94 (0.68–1.31) p = 0.722	1.32 (0.98–1.79) p = 0.066	1.01 (0.79–1.30) p = 0.934	0.94 (0.83–1.06) p = 0.299
3)mid dep.	1.07 (0.77–1.48) p = 0.708	1.16 (0.85–1.59) p = 0.346	0.81 (0.62–1.06) p = 0.119	(0.90–1.13) p = 0.902
4) 2^nd^ most	1.21 (0.86–1.69) p = 0.272	1.06 (0.76–1.47) p = 0.731	0.87 (0.67–1.13) p = 0.302	0.88 (0.78–0.98) p = 0.025
5)most dep.	0.99 (0.69–1.40) p = 0.939	1.12 (0.80–1.56) p = 0.515	0.90 (0.70–1.18) p = 0.453	(0.84–1.05) p = 0.303
Dizzy: Yes	1.25 (0.97–1.60) p = 0.086	1.70 (1.35–2.14) p<0.001	1.21 (1.00–1.47) p = 0.053	1.06 (0.87–1.29) p = 0.658
Hearing deficit: Yes	0.90 (0.67–1.22) p = 0.501	0.85 (0.64–1.13) p = 0.258	0.86 (0.78–0.96) p = 0.009	0.99 (0.81–1.21) p = 0.913
Visual deficit: Yes	1.12 (0.86–1.48) p = 0.401	1.24 (0.96–1.60) p = 0.105	0.99 (0.82–1.20) p = 0.897	0.97 (0.80–1.18) p = 0.778
CCI score				
0	Referent	Referent	Referent	Referent
1	0.78 (0.57–1.07)	0.84 (0.63–1.13)	1.09 (0.88–1.36)	0.97 (0.77–1.21)
2–8	p = 0.121 0.53 (0.32–0.87) p = 0.011	p = 0.259 0.90 (0.59–1.36) p = 0.611	p = 0.423 1.03 (0.78–1.37) p = 0.812	p = 0.778 1.16 (0.89–1.51) p = 0.279
BMI	1.03 (1.00–1.05) p = 0.023	1.00 (0.98–1.02) p = 0.988	0.99(0.98–1.00) p = 0.029	0.98 (0.96–1.00) p = 0.035
Depression	1.02 (0.97–1.06) p = 0.422	1.03 (0.99–1.07) p = 0.175	1.02 (0.99–1.06) p = 0.239	(0.98–1.05) p = 0.399
Anxiety	1.02 (0.99–1.06) p = 0.233	0.98 (0.95–1.01) p = 0.222	1.00 (0.99–1.01) p = 0.756	0.98 (0.96–1.01) p = 0.183
Cognitive complaint	1.01 (1.00–1.02) p<0.001	1.01 (1.00–1.01) p<0.001	1.00 (1.00–1.01) p = 0.058	1.00 (1.00–1.01) p = 0.052
Total medication	(1.00–1.09) p = 0.056	1.02 (0.98–1.06) p = 0.411	1.05 (1.02–1.08) p = 0.001	1.03 (1.00–1.06) p = 0.050
Analgesics				
None	Referent	Referent	Referent	Referent
Basic	1.10 (0.71–1.69) p = 0.667	0.90 (0.59–1.35) p = 0.599	1.16 (1.01–1.34) p = 0.042	1.08 (0.82–1.42) p = 0.593
Weak op.	1.23 (0.78–1.93) p = 0.366	0.94 (0.61–1.45) p = 0.782	1.03 (0.88–1.21) p = 0.686	0.93 (0.68–1.27) p = 0.661
Mod. op.	1.20 (0.78–1.83) p = 0.406	0.94 (0.61–1.45) p = 0.782	1.05 (0.91–1.21) p = 0.488	0.86 (0.62–1.18) p = 0.345
Strong op.	1.61 (1.05–2.47) p = 0.029	1.24 (0.83–1.87) p = 0.298	1.00 (0.86–1.17) p = 0.981*	1.47 (1.07–2.01) p = 0.01*
V.strong op.	1.46 (0.22–9.83) p = 0.698	0.48 (0.50–4.51) p = 0.521		
NSAIDs: Yes	0.86 (0.62–1.19) p = 0.360	1.27 (0.94–1.70) p = 0.114	(0.79–1.32) p = 0.893	0.93 (0.70–1.21) p = 0.575
Physical functioning				
No problem	Referent	Referent	Referent	Referent
A little	1.18 (0.87–1.61) p = 0.286	1.81 (1.36–2.39) p<0.001	1.04 (0.83–1.32) p = 0.698	1.15 (0.91–1.45) p = 0.239
A lot	1.10 (0.73–1.68) p = 0.647	1.91 (1.31–2.80) p = 0.001	0.80 (0.60–1.07) p = 0.140	1.09 (0.83–1.44) p = 0.555
Previous fall: Yes	2.70 (2.01–3.61) p<0.001	1.96 (1.47–2.61) p<0.001	1.51 (1.22–1.88) p<0.001	1.11 (0.88–1.39) p = 0.370

OR = odds ratios; HR = hazard ratios 95%; CI = 95% confidence interval; FT Ed >16y = continuing in full time education beyond aged 16 years; IMD = Index of Multiple Deprivation divided into quintiles, where: least dep. = least deprived, 2nd least = 2nd least deprived, mid dep. = middle deprivation category, 2nd most = 2nd most deprived, most dep. = most deprived category. Charlson Comorbidity Index (CCI) score: 0 = no CCI comorbidities, 1 = 1 CCI comorbidity, 2–8 = 2 or more CCI comorbidities. BMI = body mass index. Total medication = total medication count. Analgesics: weak op. = weak opiates, mod. op. = moderate strength opiates, strong op. = strong opiates, v. strong op. = very strong opiates; *for falls requiring primary health care and falls requiring hospitalisation, analgesic categories strong and very strong opioids are combined due to small numbers. NSAID = non steroidal anti = inflammatory drug. Physical functioning = ability to walk 100 yards: no problem = no physical limitation, a little = a little limitation, a lot = a lot of limitation in ability to walk 100 yards. Previous fall = baseline self-reported fall recorded as ‘yes’. Interaction terms with p<0.05 included in final models were: 3 year self-reported fall–none; 6 year self-reported fall–‘widespread painXhearing deficit’; fall requiring primary healthcare–‘widespread painXage, widespread painXcognitive complaint’; falls requiring hospitalisation–‘widespread painXIMD’, ‘widespread painXanxiety’, ‘widespread painXtotal medication count, ‘widespread painXphysical functioning’.

Table 3 shows an increasing number of pain sites at baseline remains associated with increased odds of a future fall at three years (OR 1.12 (1.01–1.24) p = 0.037) and six years (OR 1.02 (1.00–1.03) p = 0.035) for each unit increase in pain site number. An increasing number of pain sites at baseline was statistically significantly associated with future falls requiring primary healthcare in the unadjusted model; the adjusted model reports a HR of 1.01 ((1.00–1.02) p = 0.106). The baseline number of pain sites was not associated with future falls requiring hospital admission in either the unadjusted or adjusted analyses.

The presence of widespread pain at baseline was associated with three year self-reported fall in the unadjusted analysis (OR 2.96 (2.31–3.80) p<0.001) and the adjusted model (OR 1.27 (0.92–1.75) p = 0.143), although the relationship in the adjusted analysis did not remain statistically significant. Widespread pain at baseline was associated with increased odds of future six-year self-reported fall with the unadjusted OR 2.90 ((2.27–3.70) p<0.001) and the adjusted OR 1.43(1.06–1.95) p = 0.021). The presence of ‘some pain’ at baseline was associated with a 26% increase in risk of future falls requiring primary healthcare use (HR 1.26 (1.01–1.57) p = 0.048) and baseline widespread pain had a 27% risk of future fall requiring primary healthcare use (HR 1.27 (0.98–1.65) p = 0.094). Widespread pain at baseline was not associated with future fall requiring secondary healthcare (adjusted HR 0.98 (0.77–1.25) p = 0.907).

Each fall category has a different set of predictors. Increasing age, being female and self-reported falls at baseline is associated with all types of fall. Increasing cognitive complaint at baseline predicts self-reported falls and fall requiring primary healthcare. Increasing total medication count at baseline predicts 3 year self-reported fall, falls requiring primary healthcare and falls requiring hospital admission.

Analyses were performed using age dichotomized into adults aged 50 to 64 years and adults aged 65 years and older (S2 Table provides further detail). Differences between the age-group analyses and pooled analyses were found for self-reported fall at 3 years, where adults aged 64 years and under had a statistically significant association with the baseline number of pain sites, but the older group did not. The trend towards increasing number of pain sites and self-reported falls at 6 years remained, though the association was no longer statistically significant at p<0.05 level. When age was treated as a continuous variable, baseline widespread pain predicted 6 year self reported fall; this relationship remained in the 65 years and older age group but not for adults aged 64 years and under. In Table 3, baseline number of pain sites did not predict falls requiring primary healthcare; this association became statistically significant in adults aged 65 years and older. Table 3 presents an association between baseline widespread pain and falls requiring primary health care; this relationship did not reach statistical significance in the age-group analyses. Analysis using age categories did not reveal additional associations with falls requiring hospital admission.

Sensitivity analysis (S3 Table provides further details) found no association between the baseline number of pain sites and falls requiring primary health care; predictors for fall requiring primary health care were increasing age, sex and presence of dizziness. There was no association between baseline widespread pain and falls requiring primary healthcare; predictors of falls requiring primary health care in this model were increasing age, sex, moderate combination opioids and moderate opioid analgesics, and dizziness. The baseline number of pain sites was not associated with future fall requiring hospitalization; increasing age, sex, increasing anxiety scores, increasing total medication count and limitation in physical functioning were all predictive covariates. Widespread pain at baseline was not associated with falls requiring hospital admission; predictors of falls requiring hospital admission in this model were increasing age, sex, increasing total medication count, prescription for non-steroidal anti-inflammatory medication and limited physical functioning.

## Discussion

Measured as number of pain sites and widespreadness, baseline multisite pain predicted future self-reported fall at three and six years independent of known falls’ risk factors and putative confounders. Multisite pain at baseline independently predicted future fall requiring primary healthcare use, though this relationship was attenuated by known falls risk factors and other putative confounders. Multisite pain at baseline did not predict future fall requiring secondary healthcare admission when accounting for known falls’ risk factors and other putative confounders.

The present study develops previous observations that multisite pain predicts self-reported falls [8] by accounting for additional known fall risk factors and putative confounders during analysis; it also provides the first exploration of the relationship between multisite pain and future falls requiring primary healthcare use. Multisite pain did not predict falls requiring secondary healthcare admission, a finding supported by a Sweden-based population study exploring multisite pain and injurious falls in older women [43], however this study did find that older men reporting multisite pain were more likely to experience future injurious fall during a 10 year follow-up [43].

Although odds ratios and hazard ratios are not directly comparable, the reduction in magnitude of effect in predicting falls requiring primary healthcare use and the loss of effect in predicting falls requiring secondary healthcare admission suggests that, as falls increase in severity, other factors become stronger predictors. In general, the number of predictors decreased as fall severity increased. At the most severe end of the spectrum, falls requiring hospital admission were predicted by three covariates: advancing age, being female and reducing body mass index. These covariates are also risk factors for the development of osteoporosis [44] and thus predict fall-related fragility fractures. The predilection for fall-related fragility fractures in older women compared to older men may also explain the differences in relationship between pain and injurious falls in the Swedish-based study.

Inspecting the differences in falls risk by age-group analysis compared with pooled results, the association between baseline number of pain sites and future 3 year self-reported fall is due to the association in adults aged 50–64 years, perhaps as they have not yet accumulated additional falls risk factors in their life course and are thus more prone to fall as a direct result of pain than their older counterparts. As the study population ages there is no longer a statistically significant relationship between baseline number of pain sites and future self-reported falls at 6 years, perhaps suggesting that during this period other falls risk factors dominate falls risk. The relationship between baseline widespread pain and 6 year self-reported fall is strongest in adults aged 65 years and older, perhaps an indication that, although the number of pain sites is no longer predictive of self-reported falls as other falls risk factors accumulate, the widespread pattern of the pain is more significant than a simple pain site count in contributing to self-reported falls at 6 years in the older old age group. Age group analysis of falls requiring primary healthcare loses the association with a baseline status of ‘some pain’ and has retained the small degree of statistical significance in adults aged 65 years and older.

The sensitivity analyses confirmed no association between baseline multisite pain and falls requiring primary healthcare and hospital admission in the population with complete follow up (n = 4,386). This is different from the whole sample analysis (n = 11,375) for future falls requiring primary healthcare, where each additional number of pain sites at baseline was found to confer a 1% additional risk of and ‘some pain’ conferred an additional 26% increased risk. Furthermore, different covariates predicted future healthcare associated fall. Both of these differences may be explained by the difference between study populations. The population with complete follow up were younger, had fewer physical health problems, fewer prescribed analgesics and better physical functioning scores, all likely a consequence of the healthy cohort effect. The difference in multisite pain as a predictor of falls requiring primary healthcare may indicate that, as falls’ risk factors are accumulated through the life course, pain becomes more strongly independently predictive of future fall risk. This phenomenon may be explained by the resulting cumulative reduction in an individual’s capacity to compensate for situations that may result in a fall, such that a fall occurs. For example, the cognitive processes required to avoid falling are challenged by the presence of pain and can no longer be tempered in the context of polypharmacy, multimorbidity and limited physical functioning. These findings raise the possibility that using the presence of multisite pain as a red flag for further falls prevention is more relevant to individuals who have accumulated other falls risk factors, thus moving towards a stratified approach to falls prevention. Further research is advised to further explore this potential new approach.

This study has examined the prospective relationship between multisite pain and falls using the largest sample in published literature to date. Data linkage between survey responses, primary care electronic health records, hospital admissions data and national mortality data adds a novel methodology to the evidence base around pain and falls.

Despite a comprehensive covariate selection process, the omission of potentially important covariates due to lack of data availability is acknowledged. For example, pain intensity, which has been shown to predict future self-reported fall [8], might alter the demonstrated relationship between multisite pain and falls and it is advisable to consider including this in future research.

The self-reporting of falls relies on recall over a three month period which has been found to be problematic, particularly for recall at 3 and 6 months [45]. It is therefore possible that self-reported falls were underreported and fallers were misclassified as non-fallers, thus any association between multisite pain and falls may be underestimated. There were fewer falls requiring primary healthcare use than hospital admission during the study period. This may indicate that falls are under-recorded in electronic primary care records, perhaps due to clinicians coding consultations with the cause or consequence of the fall.

## Conclusion

Multisite pain must now be added as a falls risk factor in international guidelines to ensure clinicians identify patients at risk of falls due to pain and implement current falls prevention strategies. Exploring the impact of reducing multisite pain upon future falls risk is the next step towards developing novel falls prevention programmes to reduce the burden of falls for adults in middle and older age, their communities and wider society.

## Supporting information

S1 Table Fall related READ codes that are used to extract GP-recorded falls status. The complete list of read-codes used to extract GP-recorded fall status.
(DOCX)

S2 Table Adjusted odds ratios and hazard ratios for multisite pain and self-reported falls, falls requiring primary healthcare utilisation, and falls requiring secondary healthcare admission: Analysis by dichotomised age. (DOCX)

S3 Table Sensitivity analysis: Adjusted odds ratios and hazard ratios for multisite pain and falls requiring primary healthcare utilisation or secondary healthcare admission. (DOCX)
