# Supplementary material for: Investigating multisite pain as a predictor of self-reported falls and falls requiring health care use in an older population: A prospective cohort study
Source: PLoS One. 2019 Dec 11;14(12):e0226268. doi: 10.1371/journal.pone.0226268 (PMC6905547; doi:10.1371/journal.pone.0226268)
Supplement: S1 Table — The complete list of read-codes used to extract GP-recorded fall status. (DOCX) [file pone.0226268.s001.docx]

**S1 Table: Fall related Read Codes that are used to extract GP-recorded falls status**

| **5 byte READ code** | **Meaning** |
| --- | --- |
| 16D..  16D1.  16D2.  16D3.  16D4.  16D5. | Falls  Recurrent falls  Number of falls in the last year  Does not fall  No fear of falls  Fall onto outstretched hand |
| U10..  U100.  U101.  U102.  U103.  U104.  U105.  U106.  U107.  U108.  U109.  U10A.  U10B.  U10C.  U10D.  U10E.  U10F.  U10G.  U10H.  U10J.  U10z. | [X] Falls  [X] Fall on same level involving ice and snow  [X] Fall on same level from slipping, tripping and stumbling  [X] Fall involving ice-skates skis roller-skates or skateboards  [X] 0^th^ fall same level due collision/ pushing by another person  [X] Fall while being carried or supported by another person  [X] Fall involving wheelchair  [X] Fall involving bed  [X] Fall involving chair  [X] Fall involving other furniture  [X] Fall involving playground equipment  [X] Fall in and from stairs and steps  [X] Fall on / from ladder  [X] Fall on and from scaffolding  [X] Fall from, out of or through building or structure  [X] Fall from tree  [X] Fall from cliff  [X] Diving / jumping into water causing injury other than drowning or submersion  [X] Other fall from one level to another  [X] Other fall on same level  [X] Unspecified fall |
| TC…  TC0..  TC1..  TC2..  TC3..  TC4,,  TC5,,  TC6..  TC7..  TCy..  TCz.. | Accidental falls  Fall on or from stairs or steps  Fall on of from ladders or scaffolding  Fall from our out of building or other structure  Fall into hole or other opening in surface  Other fall from one level to another  Fall on same level from slipping, tripping or stumbling  Fall on same level– collision/push/shove by/ with other person  Fracture, cause unspecified  Other falls  Accidental falls NOS |
