# Supplementary material for: Investigating multisite pain as a predictor of self-reported falls and falls requiring health care use in an older population: A prospective cohort study
Source: PLoS One. 2019 Dec 11;14(12):e0226268. doi: 10.1371/journal.pone.0226268 (PMC6905547; doi:10.1371/journal.pone.0226268)
Supplement: S2 Table — (DOCX) [file pone.0226268.s002.docx]

**Supplementary information 2**

**Adjusted odds ratios and hazard ratios for multisite pain and self-reported falls, falls requiring primary healthcare utilisation, and falls requiring secondary healthcare admission: analysis by dichotomised age**

| Number of pain sites | Self-reported 3 y fall | Self-reported 6y fall | GP fall | HES fall |
| --- | --- | --- | --- | --- |
| Age <65 | OR 1.04 (1.02-1.06) | OR 1.02 (0.99-1.04) | HR 1.00 (0.97-1.02) | 0.99 (0.97 – 1.02) |
| Age >=65 | OR 1.01 (0.76-1.34) | OR 1.01 (0.99-1.04) | HR 1.01 (1.00-1.03) | 1.00 (0.99-1.02) |

| Widespread pain | Self-reported 3 y fall | Self-reported 6y fall | GP fall | HES fall |
| --- | --- | --- | --- | --- |
| Age <65 |  |  |  |  |
| Some pain | 1.10 (0.74.1.61) | 1.41 (0.96-2.09) | 1.34 (0.84-2.20) | 0.64 (0.02-23.84) |
| Widespread pain | 1.45 (0.95-2.20) | 1.35 (0.87-2.09) | 1.28 (0.74-2.23) | 0.58 (0.00-790.40) |
| Age >=65 |  |  |  |  |
| Some pain | 0.92 (0.59-1.44) | 1.11 (0.75-1.65) | 1.05 (0.22-5.00) | 0.95 (0.75-1.20) |
| Widespread pain | 1.16 (0.70-1.92) | 1.58 (1.01-2.45) | 0.90 (0.04-19.38) | 1.13 (0.85-1.49) |
