# Supplementary material for: Investigating multisite pain as a predictor of self-reported falls and falls requiring health care use in an older population: A prospective cohort study
Source: PLoS One. 2019 Dec 11;14(12):e0226268. doi: 10.1371/journal.pone.0226268 (PMC6905547; doi:10.1371/journal.pone.0226268)
Supplement: S3 Table — (DOCX) [file pone.0226268.s003.docx]

**Supplementary Information 3**

**Sensitivity analysis:** **adjusted odds ratios and hazard ratios for multisite pain and falls requiring primary healthcare utilisation or secondary healthcare admission**

| **Covariate** | **Falls requiring primary health care**  **n=3801**  **HR (95% CI)** | **Falls requiring hospitalisation**  **n=3801**  **HR (95% CI)** | **Falls requiring primary health care**  **n=3801**  **HR (95% CI)** | **Falls requiring hospitalisation**  **n=3801**  **HR (95% CI)** |
| --- | --- | --- | --- | --- |
| Number of pain sites | 1.00 (0.98-1.02) | 0.98 (0.96-1.01) |  |  |
| No pain | - | - | Referent | Referent |
| Some pain |  |  | 1.18 (0.82-1.70) | 0.70 (0.47-1.04) |
| Widespread |  |  | 1.09 (0.71-1.68) | 0.57 (0.35-0.93) |
| Age (years) | 1.07 (1.05-1.09) | 1.07 (1.05-1.09) | 1.07 (1.05-1.09) | 1.07 (1.04-1.09) |
| Sex: Male | 1.19 (1.01-1.39) | 0.51 (0.36-0.74) | 1.19 (1.00-1.39) | 0.51 (0.36-0.74) |
| FT Ed >16y: No | 0.82 (0.56-1.19) | 0.70 (0.45-1.10) | 0.82 (0.56-1.19) | 0.72 (0.46-1.13) |
| Income adequate | 0.99 (0.73-1.34) | 1.10 (0.78-1.56) | 0.96 (0.73-1.33) | 1.11 (0.79-1.57) |
| Occ Class non-manual | 1.08 (0.81-1.45) | 0.76 (0.54-1.08) | 1.09 (0.82-1.45) | 0.75 (0.53-1.07) |
| IMD |  |  |  |  |
| 1)least dep. | Referent | Referent | Referent | Referent |
| 2) 2^nd^ least | 1.07 (0.73-1.59) | 1.32 (0.79-2.22) | 1.08 (0.73-1.59) | 1.31 (0.78-2.20) |
| 3)mid dep. | 0.85 (0.55-1.29) | 1.05 (0.60-1.84) | 0.85 (0.56-1.29) | 1.05 (0.60-1.83) |
| 4) 2^nd^ most | 0.87 (0.56-1.35) | 1.58 (0.93-2.68) | 0.87 (0.56-1.36) | 1.55 (0.91-2.63) |
| 5)most dep. | 0.80 (0.50-1.26) | 1.34 (0.77-2.32) | 0.80 (0.50-1.26) | 1.33 (0.76-2.31) |
| Dizzy: Yes | 0.79 (0.67-0.94) | 0.96 (0.64-1.44) | 0.79 (0.68-0.94) | 0.95 (0.64-1.43) |
| Hearing deficit: Yes | 0.81 (0.55-1.19) | 1.03 (0.67-1.59) | 0.81 ((0.55-1.19) | 1.05 (0.68-1.61) |
| Visual deficit: Yes | 1.18 (0.84-1.67) | 1.09 (0.72-1.64) | 1.19 (0.85-1.67) | 1.10 (0.73-1.66) |
| CCI score |  |  |  |  |
| 0 | Referent | Referent | Referent | Referent |
| 1 | 0.85 (0.56-1.28) | 0.78 (0.48-1.26) | 0.85 (0.56-1.28) | 0.77 (0.48-1.25) |
| 2-8 | 1.44 (0.87-2.38) | 0.94 (0.50-1.79) | 1.45 (0.88-2.39) | 0.93 (0.49-1.77) |
| BMI | 1.01 (0.98-1.04) | 0.97 (0.94-1.01) | 1.01 (0.98-1.04) | 0.97 (0.76-2.31) |
| Depression | 1.01 (0.95-1.07) | 1.02 (0.95-1.09) | 1.01 (0.95-1.07) | 1.02 (0.95-1.09) |
| Anxiety | 0.99 (0.95-1.04) | 1.03 (1.00-1.04) | 0.99 (0.94-1.04) | 0.98 (0.93-1.03) |
| Cognitive complaint | 1.00 (0.99-1.01) | 1.00 (0.99-1.01) | 1.00 (0.99-1.01) | 1.00 (0.99-1.01) |
| Total medication | 1.05 (0.96-1.11) | 1.07 (1.00-1.13) | 1.05 (0.96-1.11) | 1.07 (1.00-1.14) |
| Analgesics |  |  |  |  |
| None | Referent | Referent | Referent | Referent |
| Basic | 0.74 (0.40-1.36) | 0.74 (0.38-1.46) | 0.72 (0.39-1.33) | 0.78 (0.40-1.54) |
| Weak op. | 0.98 (0.55-1.75) | 0.87 (0.44-1.73) | 0.96 (0.54-1.72) | 0.84 (0.43-1.64) |
| Mod. op. | 1.71 (1.06-2.77) | 0.87 (0.47-1.63) | 1.68 (1.04-2.73) | 0.91 (0.48-1.71) |
| Strong op. &  V.strong op. | 1.30 (0.73-1.69) | 0.37 (0.44-1.73) | 1.29 (0.72-2.03) | 0.91 (0.46-1.81) |
| NSAIDs: Yes | 1.11 (0.73-1.69) | 1.55 (0.98-2.45) | 1.11 (0.73-1.68) | 1.60 (1.01-2.54) |
| Physical functioning |  |  |  |  |
| No problem | Referent | Referent | Referent | Referent |
| A little | 0.86 (0.57-1.31) | 1.48 (0.93-2.36) | 0.86 (0.57-1.30) | 1.52 (0.95-2.42) |
| A lot | 0.63 (0.34-1.15) | 1.97 (1.07-3.63) | 0.63 (0.35-1.15) | 1.97 (1.07-3.63) |
| Previous fall: Yes | 1.42 (0.94-2.14) | 1.20 (0.73-1.97) | 1.41 (0.94-2.12) | 1.20 (0.73-1.97) |
| OR = odds ratios; HR=hazard ratios 95% ; CI = 95% confidence interval; where p<0.05, this is considered statistically significant and results are highlighted in **bold.** FT Ed >16y = continuing in full time education beyond aged 16 years; IMD= Index of Multiple Deprivation divided into quintiles, where: least dep.= least deprived, 2^nd^ least = 2^nd^ least deprived, mid dep. = middle deprivation category, 2^nd^ most = 2^nd^ most deprived, most dep. = most deprived category. Charlson Comorbidity Index (CCI) score: 0 = no CCI comorbidities, 1 = 1 CCI comorbidity, 2-8 = 2 or more CCI comorbidities. BMI = body mass index. Total medication = total medication count. Analgesics: weak op. = weak opiates, mod. op. = moderate strength opiates, strong op. = strong opiates, v. strong op. = very strong opiates; for falls requiring primary health care and falls requiring hospitalisation, analgesic categories strong and very strong opioids are combined due to small numbers. NSAID = non steroidal anti=inflammatory drug. Physical functioning = ability to walk 100 yards: no problem = no physical limitation, a little = a little limitation, a lot = a lot of limitation in ability to walk 100 yards. Previous fall = baseline self-reported fall recorded as ‘yes’. | | | | |
